# Identifying incarceration status in the electronic health record using large language models in emergency department settings

**DOI:** 10.1017/cts.2024.496

**Published:** 2024-03-11

**Authors:** Thomas Huang, Vimig Socrates, Aidan Gilson, Conrad Safranek, Ling Chi, Emily A. Wang, Lisa B. Puglisi, Cynthia Brandt, R. Andrew Taylor, Karen Wang

**Affiliations:** 1 Department of Emergency Medicine, Yale School of Medicine, New Haven, CT, USA; 2 Section for Biomedical Informatics and Data Science, Yale University School of Medicine, New Haven, CT, USA; 3 Program of Computational Biology and Bioinformatics, Yale University, New Haven, CT, USA; 4 SEICHE Center for Health and Justice, Yale School of Medicine, New Haven, CT, USA; 5 Department of Medicine, Yale School of Medicine, New Haven, CT, USA; 6 Equity Research and Innovation Center, Yale School of Medicine, Yale University, New Haven, CT, USA

**Keywords:** ChatGPT, emergency department, incarceration, justice involvement, large language models, machine learning, natural language processing

## Abstract

**Background:**

Incarceration is a significant social determinant of health, contributing to high morbidity, mortality, and racialized health inequities. However, incarceration status is largely invisible to health services research due to inadequate clinical electronic health record (EHR) capture. This study aims to develop, train, and validate natural language processing (NLP) techniques to more effectively identify incarceration status in the EHR.

**Methods:**

The study population consisted of adult patients (≥ 18 y.o.) who presented to the emergency department between June 2013 and August 2021. The EHR database was filtered for notes for specific incarceration-related terms, and then a random selection of 1,000 notes was annotated for incarceration and further stratified into specific statuses of prior history, recent, and current incarceration. For NLP model development, 80% of the notes were used to train the Longformer-based and RoBERTa algorithms. The remaining 20% of the notes underwent analysis with GPT-4.

**Results:**

There were 849 unique patients across 989 visits in the 1000 annotated notes. Manual annotation revealed that 559 of 1000 notes (55.9%) contained evidence of incarceration history. ICD-10 code (sensitivity: 4.8%, specificity: 99.1%, F1-score: 0.09) demonstrated inferior performance to RoBERTa NLP (sensitivity: 78.6%, specificity: 73.3%, F1-score: 0.79), Longformer NLP (sensitivity: 94.6%, specificity: 87.5%, F1-score: 0.93), and GPT-4 (sensitivity: 100%, specificity: 61.1%, F1-score: 0.86).

**Conclusions:**

Our advanced NLP models demonstrate a high degree of accuracy in identifying incarceration status from clinical notes. Further research is needed to explore their scaled implementation in population health initiatives and assess their potential to mitigate health disparities through tailored system interventions.

## Introduction

Perhaps one of the most underappreciated but highly prevalent social determinants of health is being exposed to incarceration. The United States has one of the highest incarceration rates globally, with over 7 million admissions to jails annually and over 1.2 million in prison as of year-end 2022 [1–3]. Disproportionately high incarceration rates are observed among racially minoritized individuals, as well as those of low socioeconomic status. Incarcerated individuals have higher rates of communicable and noncommunicable diseases, in addition to mental health and substance use disorders compared with those never incarcerated [4,5]. It is estimated that 40 percent of these individuals receive their diagnoses while incarcerated, where there is a constitutional guarantee to health care, but where the acquisition of self-management skills for chronic diseases is hindered by the restrictive and punitive nature of the penal system [6].

Upon release, these individuals continue to encounter barriers to care, including limited access to housing, employment, and primary care services [7,8]. Compounding these issues, inadequate coordination of care transitions between correctional facilities and community health systems contributes to an elevated risk of death, hospitalization, and deteriorating health outcomes post-release [9]. Past work indicates that people with histories of incarceration face significant barriers to accessing consistent and high-quality care, including under-insurance and discrimination within the healthcare system [10–12].

These underlying structural factors and social needs drive an important association between increased frequency of acute care utilization and recent or impending incarceration. Studies have revealed a correlation between the frequency of Emergency Department (ED) visits and subsequent jail encounters within a year. Particularly, individuals with super-frequent ED usage, defined as 18 or more visits per year, are found to have 12.3 times higher odds of subsequent incarceration. In addition, those who were incarcerated saw a significantly increased likelihood of visiting the ED within 30 days prior to incarceration or 30 days following jail exit [13]. These interactions with the health system serve as opportunities for health system level interventions to address this social risk, such as engagement in interventions to prevent incarceration (initiation of medications for opioid use disorder, violence intervention programs) or prevent poor outcomes after release (engagement into primary care programs)[14–17]. Additionally, systematically implementing broader health systems level interventions, such as medical legal partnerships, and quality of care analyses necessitate an ability to identify those with a history of incarceration within health system information systems. Currently there is no reliable way to do collect information on this social determinant of health and screening directly can be stigmatizing [18,19].

The electronic health record (EHR) holds promise as a research tool for understanding the drivers of poor health among individuals with a history of incarceration, given the large sample sizes, generalizability to a wide range of patient populations, low expense, and relatively fewer resources needed to conduct studies [19]. However, EHRs currently are not designed to systematically measure incarceration exposure. Providers do not receive training in how to ask about or consistently document incarceration history into patients' social history, leading to current limitations in the documentation of incarceration history in standardized or structured formats [18]. Natural language processing (NLP) has the potential to extract valuable information from unstructured data in the EHR, such as in provider notes. NLP techniques, such as named entity recognition, relation extraction, and text classification, can identify relevant information and classify clinical notes according to specific criteria. So far, studies that examine the EHR’s ability to accurately capture data regarding incarceration exposure are limited but demonstrate the potential of this approach. One previous study assessed the identification of incarceration history using an NLP tool, YTEX, on a dataset created through linkage of Veterans’ Health Affairs (VHA) EHR, the Department of Correction (DOC) data, and Centers of Medicare and Medicaid Services (CMS) data. While findings were promising for NLP as an effective means of identification of incarceration history, the study was limited to only VHA EHR which is not generalizable to other health system EHRs. In addition, the YTEX NLP tool is an example of a rule-based NLP in comparison to deep learning techniques for NLP that are able to handle the variability and diversity of human language better in settings utilizing unstructured data, such as clinician notes from the ED [19]. Boch *et al*. proposed a BERT-based model that examined overall parental justice involvement among the pediatric population, demonstrating the utility of NLP in the identification and exploration of justice involvement [20]. However, there currently is no generalized tool which identifies an individual’s own history of incarceration and timing of the event based on unstructured clinical encounter notes.

The primary objective of this investigation was to develop an accurate NLP model, using state-of-the-art methods, to reliably and accurately identify incarceration history from unstructured clinical notes in the EHR. We also aimed to assess the ability for other large language models, such as Generative Pre-trained Transformer 4 (GPT-4), in its performance of identifying incarceration status. By pursuing these goals, our investigation will contribute to a better understanding of the utility of NLP techniques for identifying incarceration history in the EHR context, paving the way for improved research on the health of individuals with a history of incarceration and the development of targeted interventions to address their unique health needs.

## Material and methods

### Study population and setting

**Figure 1.**
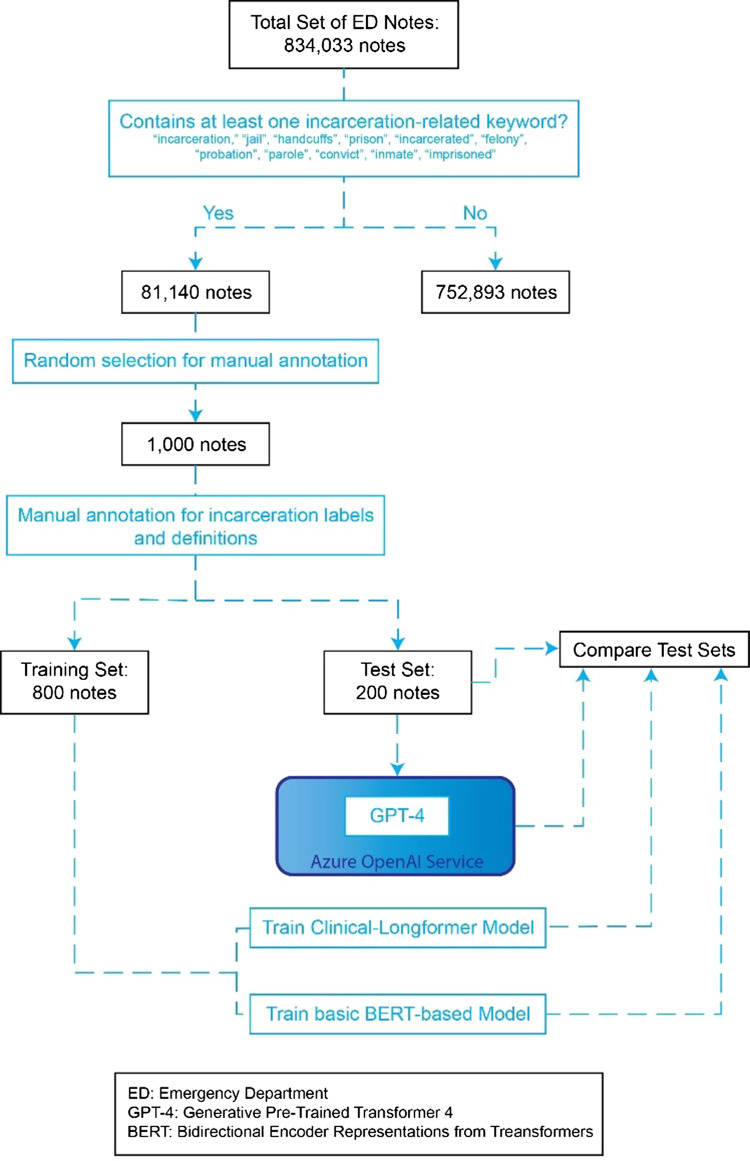
Graphic overview of note selection and NLP training process.

The study population consisted of a set of adult patients (≥ 18 years of age) who presented to the emergency department (ED) between June 2013 and August 2021 and had an ED note containing at least one of the following incarceration-related terms: “incarceration,” “jail,” “handcuffs,” “prison,” “incarcerated,” “felony,” “probation,” “parole,” “convict,” “inmate,” “imprisoned.” (Fig. 1) These terms were defined and selected after a literature review and consultation with expert opinions (LP, EW, KW, RAT). The study was completed across 10 EDs within a regional healthcare network in the northeastern United States, covering a geographic area of approximately 650 square miles, and closely resembling the overall national population [21]. The study followed the STROBE reporting guidelines for observational studies and was approved by the institutional review board, which waived the need for informed consent (HIC# 1602017249).

### Data collection and processing

From an initial set of 81,140 total clinical notes that had at least one of the prespecified key-words, we randomly sampled 1000 notes for annotation, which came from 849 unique patients across 989 visits. The size of this random sample of clinical notes was selected to ensure representation of the diverse presentations and encounter types that a patient with incarceration history could present to the ED with. To ensure model robustness to note type, a total of 25 different note types were selected, the majority of which were ED Provider Notes, Progress Notes, and ED Psych Eval Notes. A full list is in Appendix A. All text was sampled from the system-wide electronic health record (Epic, Verona, WI) using a centralized data warehouse (Helix).

### Defining history of incarceration

The broad definition of incarceration, as the state of being confined in prison or imprisonment, was further stratified into more specific statuses of previous history of incarceration, recent incarceration, and current incarceration. Similar to the process for identifying initial incarceration-related terms, related terms were chosen after an extensive literature review and consultation with expert opinions (LP, EW, KW, RAT). We stratified temporal relationship to incarceration because there are different health risks associated with each. As an example, transition into and out of correctional facilities is disruptive and traumatic and can have differential effects on health [22]. Additionally, there are likely different health system level interventions that are feasible to improve care for currently and formerly incarcerated individuals due to the role of departments of corrections in managing health care. Currently, two ICD-10 codes (Z65.1 Imprisonment and other incarceration, Z65.2 Problems related to release from prison) exist in the EHR specific to incarceration history.

### Document annotation

The process began with the assembly of a set of provider notes, capturing various clinical encounters related to incarceration and justice involvement. Using the definitions in section “Defining History of Incarceration,” senior authors (AT, KW) defined an initial set of annotation guidelines to determine incarceration status as at least one of three categories: Prior, Current, and Recent incarceration (Table 1). Led by AT, our team of annotators (LC, TH, CS) underwent thorough training on the annotation guidelines. Our annotation process then followed an iterative approach, updating guidelines while classifying an initial set of 50 notes, utilizing Fleiss’ Kappa to evaluate consistency across annotators to ensure a reliable and standardized annotation process throughout the study. Following high reliability between annotators, the remainder of the 1000 notes were randomly distributed among the annotators, and the full set was annotated. The task was framed as a classic multilabel text classification task, allowing annotators to select if patient reports had evidence of any of the following: Prior, Current, and Recent Incarceration. If a patient had a history of incarceration and was currently incarcerated, both could be selected.

**Table 1.** Incarceration history and annotation labels and their definitions

Label	Definition
Prior History of Incarceration	Patient has stated history of incarceration (jail or prison), including recent incarceration.
	Patient has stated current parole status or has a stated history of parole.
	Patient is stated to be currently living in halfway house specific to incarceration.
	Note: Label if there is evidence of history of or current parole as well as halfway houses that were explicitly mentioned to serve previously incarcerated individuals
Current Incarceration	Patient is stated to be currently in or came from jail or prison.
Recent Incarceration	Patient has stated history of being released from jail or prison in the last 6 months.
	Provider explicitly states recent release from jail or prison.

Label text classification for “Prior History” was contingent on explicit stated evidence in the note for history of incarceration, or other mentions that could allow for inference that the subject of the note had previously experienced incarceration. This included evidence of history of or current parole as well as halfway houses that were explicitly mentioned to serve previously incarcerated individuals. “Current Incarceration” was coded in instances with confirmed evidence in the text that the subject of the note came directly from a correctional facility. “Recent incarceration” required mention by the author of note stating recent release from a correctional facility or if the language was absent, an explicit mention of date of release as well as date of the note or time since release that fell within 6 months. For annotation, we employed Prodigy v1.11.7., a scriptable annotation tool designed to enable data scientists to perform the annotation tasks themselves and facilitating rapid iterative development in NLP projects.

### NLP development

Once annotations were completed, we initially fine-tuned RoBERTa, a classic BERT-based model, to predict incarceration status in ED notes using Huggingface transformers v4.20.1. However, upon initial inspection, we found that the majority of documents (68.2%) were too long to fit in the context window of classic BERT models (512 tokens, ∼400 words), reducing performance (shown in Appendix B). Therefore, we utilized an advanced BERT-based model, known as Clinical-Longformer (shown in Appendix C). Transformer-based models leverage self-attention to consider context along the full length of the input sequence. While this provides significant performance improvements, memory consumption enlarges quadratically with sequence length, making analysis of longer documents with classic transformer-based, such as BERT, models computationally infeasible. The Clinical-Longformer model uses sparse attention with a sliding context window, along with reduced global attention for key tokens to reduce memory consumption while keeping performance high and increasing context windows. In particular, we take advantage of the benefits of fine-tuning on domain-specific data and fine-tune Clinical-Longformer on our annotated incarceration status dataset [23]. We trained both the classic BERT-based model and Clinical-Longformer model on 800 notes (80%) of the data and evaluated performance on 200 (20%) notes. The models were fine-tuned to predict the presence of any of the categories of incarceration status using a multilabel classification layer added to the top of the base model. We used a binary cross-entropy loss function for training. The training process involved 10 iterations over the dataset with a predefined batch size of 16, and gradient descent optimization was utilized to minimize the loss function.

In order to measure the ability of the model to identify incarceration status generally, we collapsed the 3 labels of prior history, current, and recent history of incarceration, to represent any indication of incarceration history. For both settings, we report standard evaluation metrics such as precision, recall, and F1-score to quantitatively measure the model’s performance in identifying incarceration status from the provider notes.

### Initial prompt development and GPT-4 evaluation

**Figure 2.**
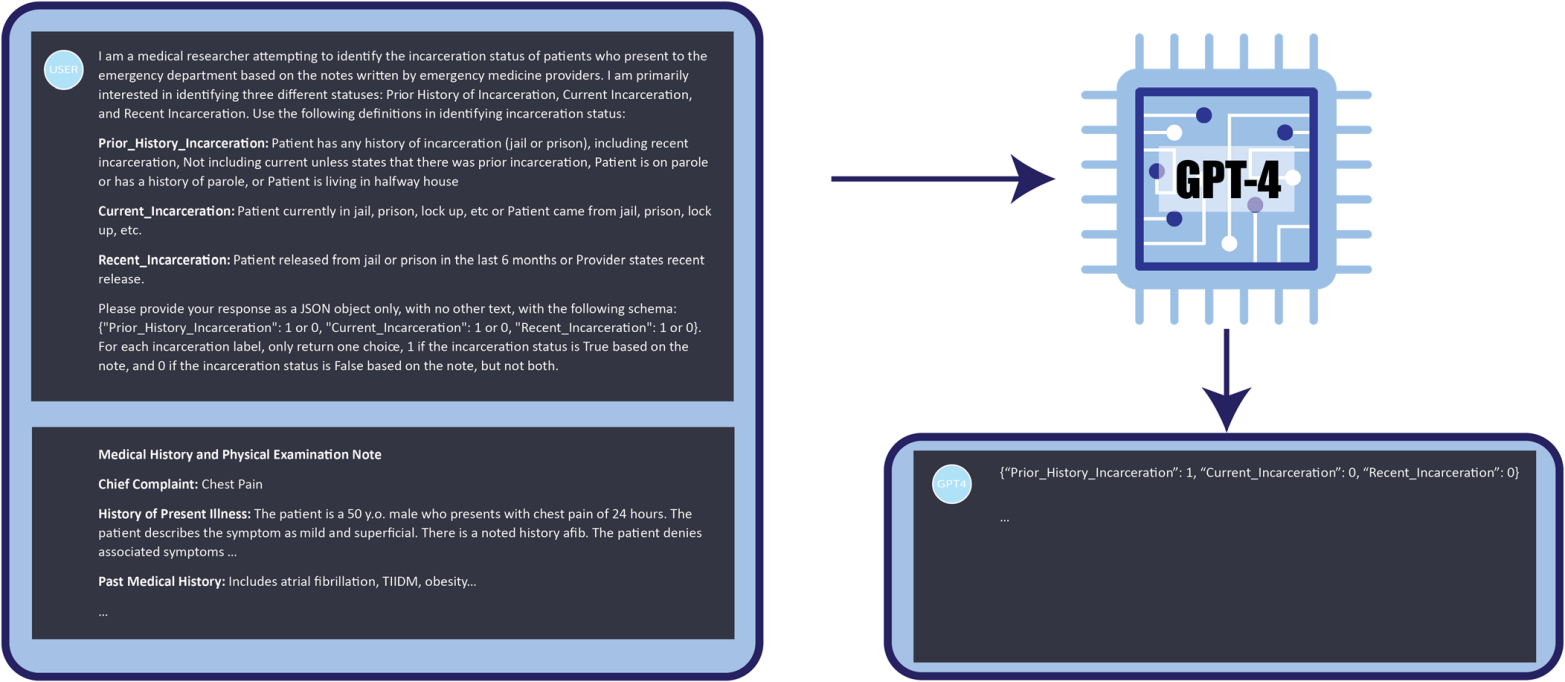
Identification of incarceration status prompt and pipeline into GPT-4 through the Azure openAI service.

Initial drafts of the prompt were deployed in the pipeline on synthetic notes external to the test set, with a total of three iterative improvements to the prompt, integrating the exact definitions used in the final iteration of the codebook for Prior History of Incarceration, Current Incarceration, and Recent Incarceration as used by the annotators when annotating the gold standard (Table 1). The final developed prompt (Fig. 2) was combined with each individual note from the test set, incorporating features of prompt design from other research, such as triple delimiters to improve recognition of parsing of input and forced JSON response to control the output.

The pipeline was deployed in the Azure OpenAI service, iterating through the entire test set, querying and retrieving JSON objects from GPT-4 that represent encoding of annotations within the three pre-defined incarceration status labels. Azure GPT-4 Model’s max input window is 8192 tokens, which was not a limitation in any instance of this test set. Within the 200-note test set, 3 instances in which notes queried to GPT-4 on the Azure OpenAI service did not receive an eligible response due to its content filtering system, such as for instances of hate, sexual, violence, and self-harm categories. These instances were omitted from the final analysis of the GPT-4 model performance as compared to gold standard rather than censoring portions of text that may be triggering the content filtering policy as such language may be important in the consideration of incarceration status.

Individual JSON objects returned by GPT-4 were combined into a python DataFrame, and the 3 labels of prior history, current, and recent history of incarceration were collapsed to an additional label of any indication of incarceration history similarly as done in the Longformer and RoBERTa models, external to the GPT-4 query.

## Results

### Dataset

**Figure 3.**
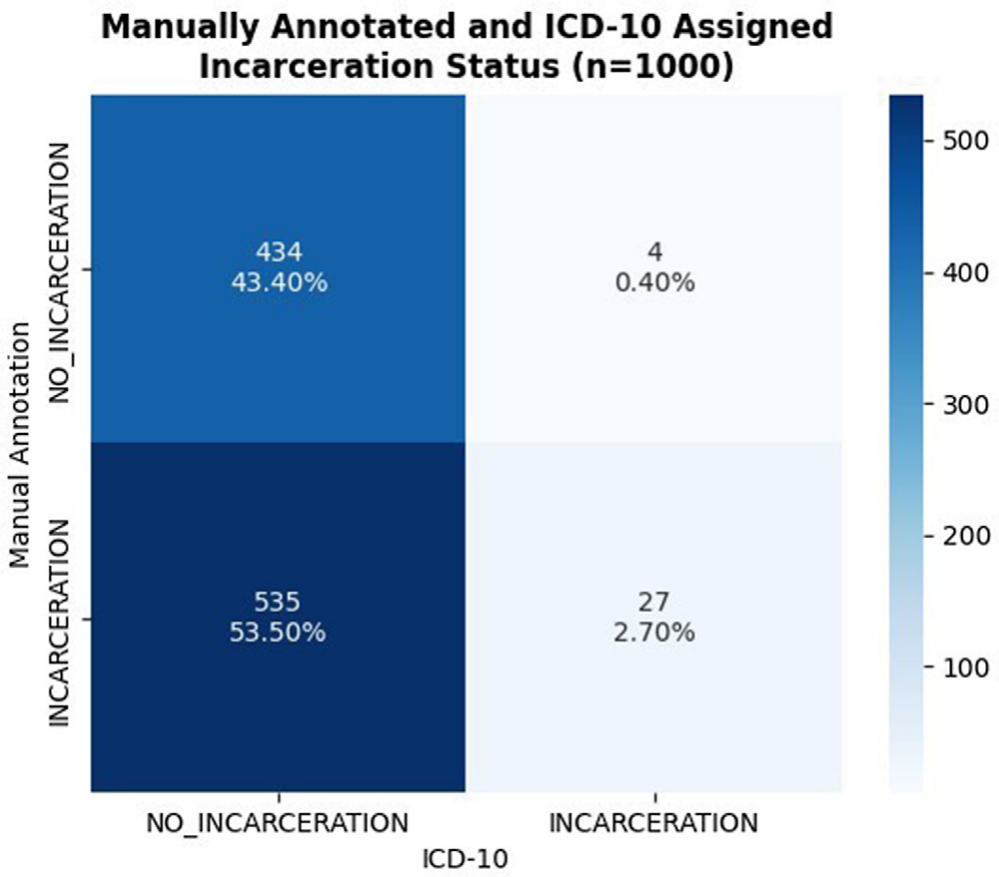
ICD-10 code vs. manual annotation.

Of the 1000 notes included which were identified as having at least one incarcerated-related term via keyword, only 559 were found to contain evidence that the patient experienced any history of incarceration, including recent incarceration (137 notes), current incarceration (80 notes), and prior history of incarceration (484 notes). Many notes that were included by simple keyword search for incarceration-related terms but not defined as containing evidence for any history of incarceration included instances where family history of incarceration was documented in the note, other forms of justice involvement, incorrect contexts such as “incarcerated hernia,” and many other examples. Utilizing ICD codes (Z65.1 Imprisonment and other incarceration, Z65.2 Problems related to release from prison) as a means of identification, only 27 of the 562 notes annotated to have any history of incarceration were identified resulting in an accuracy of 46.10%, sensitivity of 4.80%, specificity of 99.09%, precision of 87.10%, and F1 of 0.09 (Fig. 3).

### Inter-rater reliability/annotator performance

To assess the inter-rater reliability, a Fleiss’ Kappa was calculated utilizing overlap of sets of 50 annotated notes between each of the three annotators (TH, CS, LC). The annotators achieved agreement throughout annotating tasks with kappa’s of 0.826 between all annotators.

### RoBERTa natural language processing

**Figure 4.**
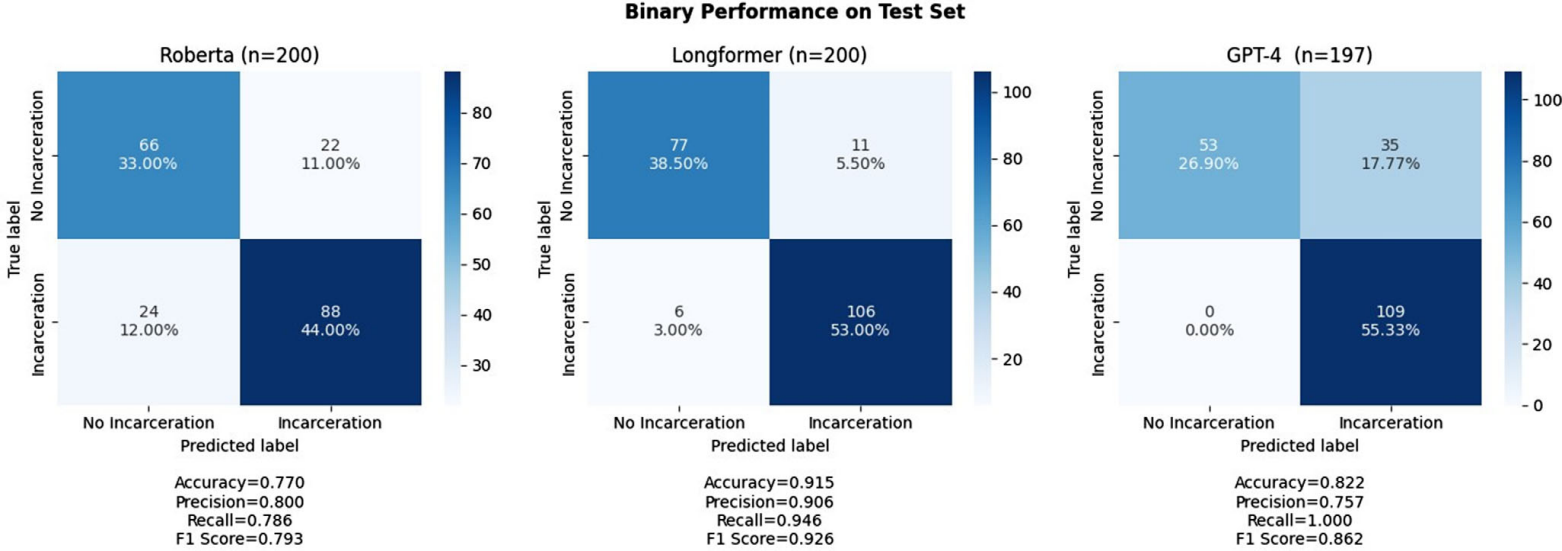
Longformer, roBERTa, and GPT-4 predicted label vs. true label by manual annotation for any history of incarceration.

To establish a baseline and point of comparison for the Clinical-Longformer model, RoBERTa, another deep learning NLP model, was utilized to identify prior history of incarceration in the test set of 200 manually identified ED encounter notes, recent incarceration, and current incarceration as well as the overall collapsed label of any history of incarceration. For the collapsed label of any history of incarceration, RoBERTa demonstrated an accuracy of 77.0%, sensitivity of 78.6%, specificity of 73.3%, precision of 80.0%, and F1 score of 0.793. Figure 4 shows three confusion matrices illustrating the performance of the RoBERTa model (left), Longformer model (middle), and GPT-4 (right). Of the total test set of 200 manually annotated notes, there were 22 encounter notes falsely labeled as positive for incarceration history and 24 falsely labeled as negative for incarceration history (Fig. 4).

**Figure 5.**
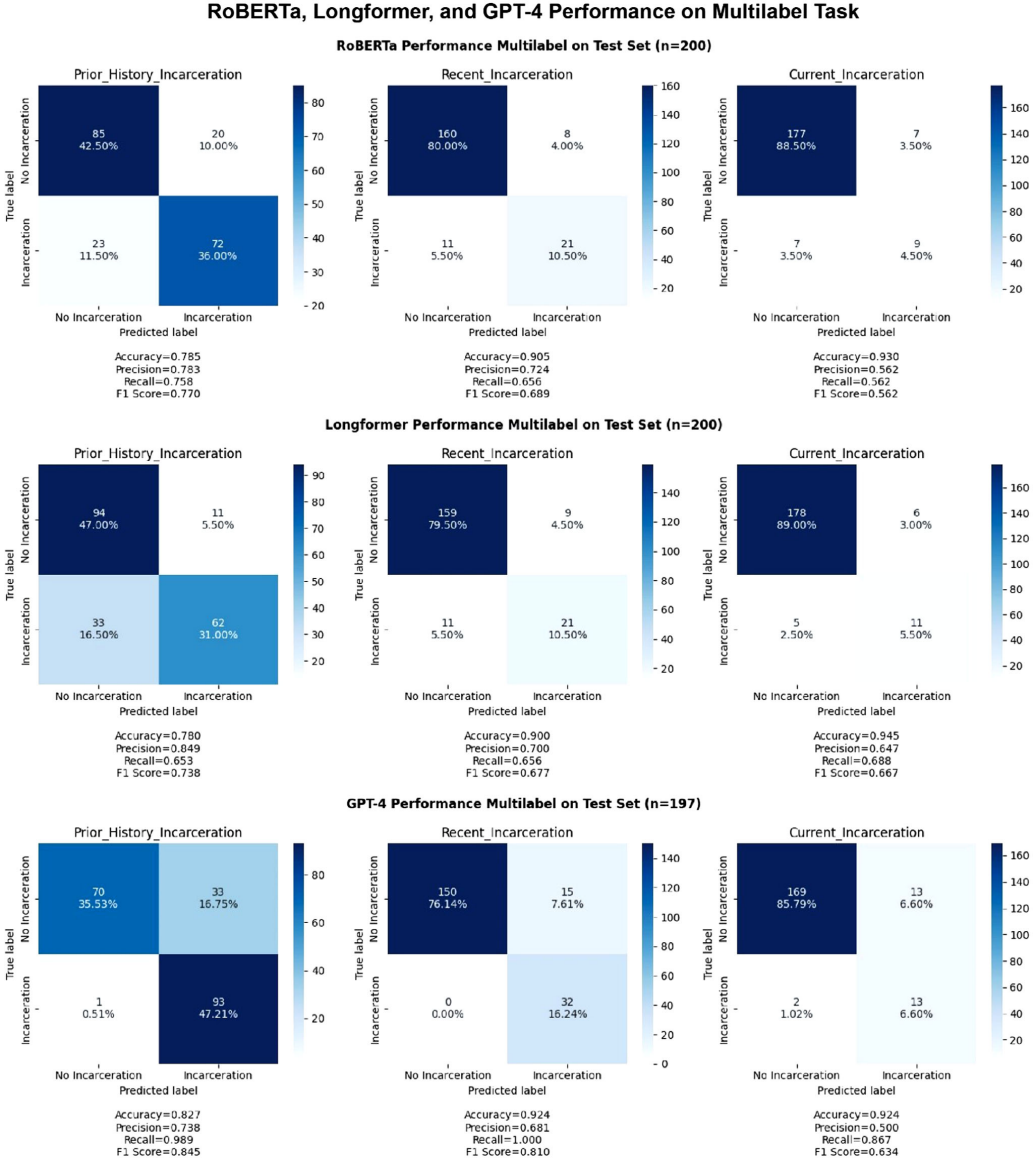
RoBERTa, clinical-longformer, and GPT-4 performance on multilabel task (prior history of incarceration, recent incarceration, and current incarceration).

As for the more specific temporal labels, RoBERTa demonstrated precision of 78.3%, recall of 75.8%, and F1 score of 0.77 for prior history of incarceration; a precision of 72.4%, recall of 65.6%, and F1-score of 0.689 for recent incarceration, and precision of 56.2%, recall of 56.2%, and F1 score of 0.562 for current incarceration (Fig. 5). Additional information about RoBERTa multilabel performance can be found in Appendix B.

### Clinical-Longformer Natural Language Processing

On the same test set of 200 manually annotated notes, the Clinical-Longformer model demonstrated an accuracy of 91.5%, sensitivity of 94.6%, specificity of 87.5%, precision of 90.6%, and F1 score of 0.926 for the identification of any history of incarceration. Of the total 200 individual test encounter notes, 11 notes were falsely identified for a positive incarceration history, and 6 notes were falsely identified as negative for incarceration history (Fig. 4).

Similar to the RoBERTa pattern of performance, the Clinical-Longformer model was relatively limited in its ability to identify specific temporal relationships and in distinguishing between prior history of incarceration (precision: 84.9%, recall: 65.3%, F1: 0.738) recent incarceration (precision: 70%, recall: 65.6%, F1: 0.677), and current incarceration (precision: 64.7%, recall: 68.8%, F1: 0.667) (Fig. 5). Additional information about Clinical-Longformer performance on the multilabel task can be found in Appendix C.

**Figure 6.**
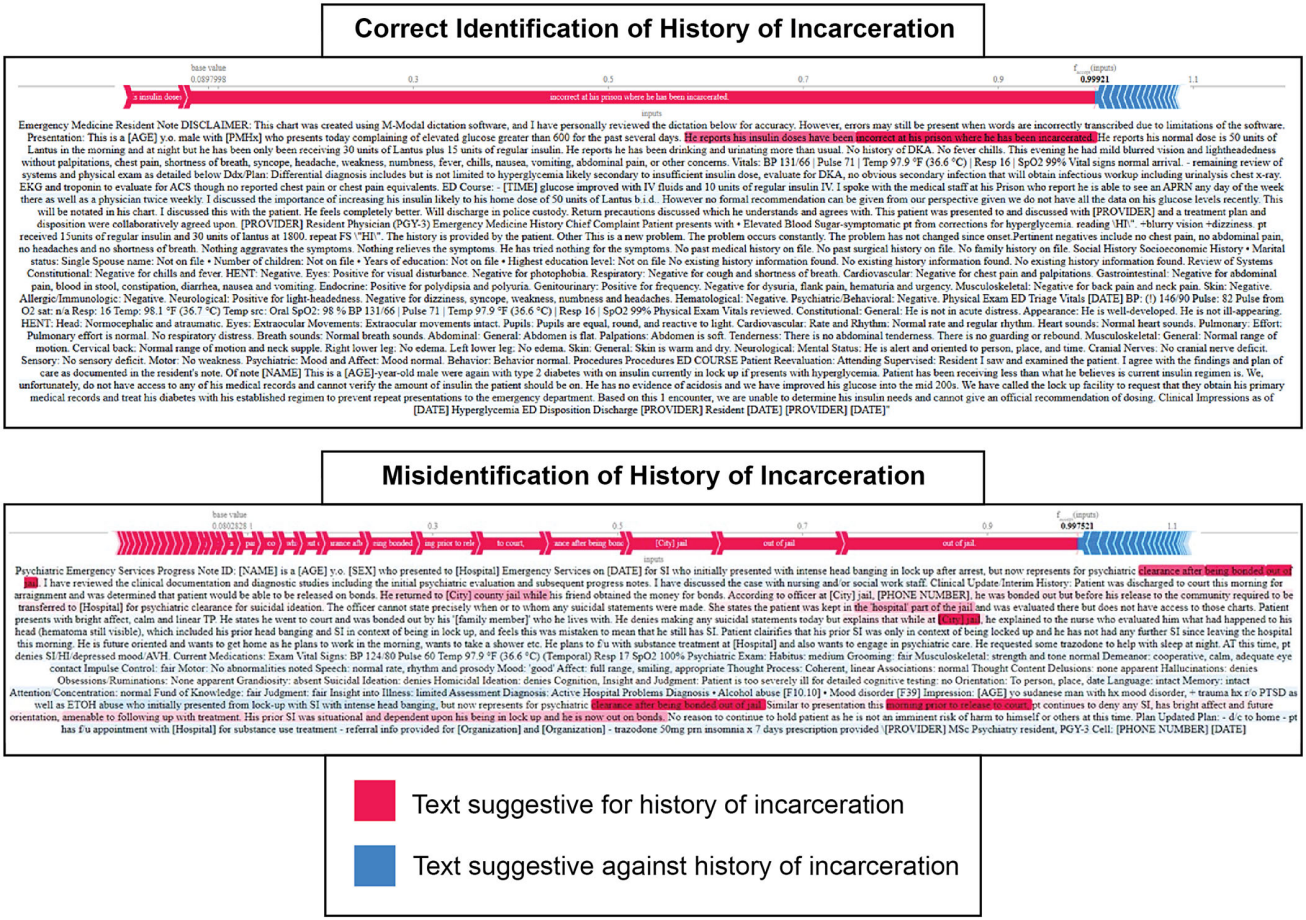
Shapley visualization of the Clinical-Longformer model correctly identifying and misidentifying any history of incarceration.

The behavior of the Clinical-Longformer model was qualitatively assessed through the use of Shapley plots to identify what contextual clues and phrases the model utilizes as signals when identifying incarceration history. These Shapley plots demonstrate tremendous utility for both assessing what elements of an ED encounter notes strongly signal to the model whether a note is positive for incarceration history or negative for incarceration history. These plots are also useful for identifying potential patterns that can cause misidentification, leading to false positives and negatives. This deidentified Shapley plot of an ED encounter note (Fig. 6) demonstrates the Clinical-Longformer model correctly identifying incarceration status. Phrases or lines of text that the Clinical-Longformer model often attends to when identifying incarceration history include “in jail,” “in prison,” “released from jail,” “when incarcerated,” “history of being incarcerated.” An interesting pattern of reporting incarceration is when it is used as a time frame, by either the patient or the physician, when discussing illness, medication usage, substance usage, such as “He reports his insulin doses have been incorrect at his prison where he has been incarcerated.”

Among those 11 notes that were false positives and 6 false negatives for incarceration history, a common trend for the Clinical-Longformer model’s confusion was complex language and phrasing separating current incarceration and instances where the individual was brought in by police or under custody, but not currently incarcerated. While often difficult to even manually annotate, the separation between instances where patients are brought in under a Police Emergency Examination Request (PEER) or from a temporary overnight lock-up is an important difference to distinguish from a patient transported from the carceral system. Phrases such as “She states the patient was kept in the “hospital” part of the jail” confused the model, causing it to be oversensitive in this regard when unable to infer the appropriate context. Other instances of oversensitivity include contextual phrases of “conviction” or “release from court.” This phrasing signals general justice involvement but not necessarily incarceration (Fig. 6).

### GPT-4 in Azure openAI service performance

In addition, the same 200 test set was queried to GPT-4 using the Azure OpenAI service to protect deidentified patient information. GPT-4, demonstrating a zero-shot approach, achieved an accuracy of 82.3%, sensitivity of 100%, specificity of 61.1%, precision of 75.7%, and F1 score of 0.86 for the identification of any history of incarceration (Fig. 4). GPT-4 was more sensitive compared to both the Longformer and RoBERTa models, but less accurate and precise than Longformer. GPT-4 demonstrated 0 false negatives, but its greater sensitivity resulted in significantly more false positives, 35 compared to the 11 of Longformer and 22 of RoBERTa NLPs models. It is important to note that GPT-4’s performance statistics omitted 3 of the 200 notes used in the test set due to the content filtering policy in-place by Azure OpenAI resulting in an inability to query an appropriate response from GPT-4.

Contrary to both the Longformer and RoBERTa patterns of performance, GPT-4 demonstrated better performance (F1 score comparison) in identifying specific temporal relationships. However, in the identification of the collapsed label of any history of incarceration, Clinical-Longformer still outpaced GPT-4, despite GPT-4’s relatively superior performance across all three temporal labels of prior history of incarceration (precision: 73.8%, recall: 98.9%, F1: 0.85), recent incarceration (precision: 68.1%, recall: 100%, F1: 0.81), and current incarceration (precision: 50%, recall: 89.7%, F1: 0.63) (Fig. 5).

## Discussion

The criminal justice system, and thus incarceration, is one of the greatest drivers of health inequity that impacts communities across the US [24]. Identifying patients with incarceration history within healthcare settings is a key initial step in attending to healthcare inequality and disparity in this underserved patient population. Our Clinical-Longformer model can reliably and accurately identify incarceration status based on free-form clinician notes in the EHR. This method offers several advantages over other forms of identification such as ICD-10 codes, rule-based NLP techniques, and other NLP techniques like RoBERTa. The NLP algorithm does not rely on providers entering ICD-10 codes which are not used accurately or reliably to measure social determinants of health. The NLP algorithm can capture nuanced information beyond these specific codes through the use of unstructured data when structured data, such as ICD-10 codes and problem lists, often under-report. In addition, the NLP algorithm surpasses simple keyword searches by considering the context and meaning of the text, leading to more accurate identification of incarceration history [25]. The Clinical-Longformer model demonstrated superior sensitivity, specificity, precision, and F1 score when compared to the RoBERTa model and a superior F1 score when compared to zero-shot GPT-4. However, when identifying specific incarceration status and further granulating any incarceration history into prior history of incarceration and recent incarceration, GPT-4 demonstrated superior performance compared to both Clinical-Longformer and the RoBERTa models.

The Clinical-Longformer model developed in this study utilizing deep learning elements offers improvement over previous methods of identification such as the rule-based YTEX model (F1: 0.75), specifically in sensitivity and overall F1 score [19]. Additionally, the utilization of a larger training set of 800 unique clinician notes compared to the 228 used in Wang et al., as well as the use of the Clinical-Longformer to improve the attention and analysis over longer notes, likely contributed to the improvement in this NLP model. The improved performance of the Clinical-Longformer model as compared to GPT-4 when defined by the F1 score is likely due to the sacrifice of sensitivity for improved precision. GPT-4 demonstrated near 100% sensitivity in recent history and prior history of incarceration labels, as well as 100% sensitivity for any history of incarceration, but significantly lower specificity compared to Clinical Longformer (60.1% vs. 87.5%)

Further, our study applies the similar principles utilized by Boch et al. to identify parental criminal justice system involvement in a pediatric population to a more specific and different goal [25]. We focused on the identification of incarceration status and history in the subject of the encounter notes, while Boch et al. looked at any pediatric exposure to parental justice involvement, including jail, prison, parole, and probation. We focused on narrowing the identification to incarceration history of the patient rather than any justice involvement, further developing our understanding of how NLP can be an asset to healthcare as populations exposed to long-term jail and incarceration histories have unique experiences, health outcomes, and possible interventions through social programs and referrals available to them.

In addition, our Clinical-Longformer model is able to capture and attend to longer documents compared to the BERT model which was used in that study, which is not able to accurately attend to notes over 500 tokens and required significant preprocessing to reduce notes down to snippets containing a total 500 tokens. A study of over 1.6 million ED provider notes, which represented a significant portion (46.2%) of the notes we used for our model, were shown to have an average of 2067 words [26]. It is important to note that one token represents 4 characters in English. Thus, the Clinical-Longformer is able to attend well to lengthy ED provider notes and other forms of unstructured data without extensive preprocessing and possible loss of important contextual information that was necessary for the original BERT-based model. GPT-4’s increased query window of 8192 tokens, as well as the availability of a 32k window, further adds to the potential of increased contextual attention if the use case arises. Although the metrics of our Clinical-Longformer model are on par with the previous work of Boch et al., the granulation of incarceration history as well as identifying incarceration history specific to the subject of the clinical encounter note can distinguish and help increase the specificity of possible utility for research purposes and possible interventions.

The use of Clinical-Longformer allows for the rapid identification of documented incarceration exposure in the EHR and may have implications for health service research, clinical care, and health outcomes for this population.

The identification of individuals who have had contact with the carceral system in EHR is an important step in understanding and mitigating disparities in healthcare and health outcomes for this population. Previous research has been limited by the difficulty of correctly identifying this population. The use of NLP as a rapid and reliable mechanism to achieve this critical step opens the possibility of future research studies targeting issues such as the disproportionate mortality rate for those diagnosed with cancer during incarceration, or the elevated cardiovascular-related morbidity and mortality for those who have been exposed to the carceral system, and providing an opportunity to study quality of care delivery [28,29]. An effective means of identifying individuals that have had contact with the carceral system via the EHR can contribute to a more comprehensive understanding of a patient’s social determinants of health and improve access to real-time referrals to social programs aimed at enhancing healthcare outcomes and finding alternative means of rehabilitation. It can also be used to help guide future research on the potential impact of incarceration on various health outcomes. Through the development of models that can help with incarceration history, steps toward improving the quality of healthcare for previously incarcerated patients can be taken as well as addressing existing disparities [27].

The utility of correctly identifying those who have been incarcerated extends beyond research or academic interests and has implications for clinical care. While currently incarcerated patients may be most easily identified in a clinical encounter, those with recent or past history of incarceration often go unidentified, as demonstrated by the poor sensitivity of current implemented systems of identification in ICD-19 codes. Such individuals could benefit by being connected to programs such as the Jail Diversion Task Force, which can help prevent incarceration or re-incarceration and offers rehabilitation to those who would benefit. The Transitions Clinic Network is another evidence-based program available in certain states that acts as a community-based primary care clinic for those returning from incarceration [30]. The use of NLP to identify our target population can improve the referral to these programs, as well as encourage the development of additional targeted interventions to help patients avoid imprisonment or reduce the impact of imprisonment on their health. However, the possibility of false positives must also be considered. Care should be taken when approaching patients, no matter how well-intentioned a provider may be, to confirm incarceration history in a non-judgmental way, and to qualify why the provider is asking, before offering resources in order to avoid eroding the patient–physician relationship by contributing to stigma.

While the NLP and machine learning approach for identifying incarceration status shows promise, it is essential to acknowledge its limitations. These limitations include data quality issues, variations in clinician note quality, and potential biases inherent in the algorithm. In addition, the standard for measurement of identification by ICD-10 codes, RoBERTa, and Clinical-Longformer is the compiled manual annotation of three different annotators under the consultation and by the definition developed by both literature review and expert opinion (RAT, KW). Although significant effort and steps were taken to ensure the standard of comparison was representative and consistently applicable, only a total of 1,000 ED encounter notes were manually annotated with a good but not perfect measure of inter-rater reliability. This represents the complexities found within the encounter notes and language when interpreting incarceration status and history.

Hesitancy by patients to disclose incarceration history, as well as hesitancy by providers to include this information in their notes, can lead to underreporting of important incarceration information, rendering the NLP unable to correctly identify incarceration history. Such hesitancy by patients in reporting incarceration history should be heavily considered when utilizing models such as our Clinical-Longformer for identifying patients with incarceration history and applying it in clinical settings. Stigma around incarceration history that is pervasive both within the healthcare system and throughout society at large. The possibility of mis-use of this incredibly powerful tool cannot be ignored. Care should be taken to limit access to this information to those who can be entrusted to work in the patient’s best interest. Thus, the Clinical-Longformer model, given its superior sensitivity and relatively poorer specificity compared to previous models, would more appropriately act as a screening tool or “potential” cohort identifier for further investigation of incarceration history rather than an endpoint of status. Manual confirmation following the use of this Clinical-Longformer model would be best to avoid false positives or misplacements of such electronic labels in a patient file.

In addition, our Clinical-Longformer model was trained over only a small subset of possible ED notes taken from a specific region of the US. While the subset and the annotation were meant to represent the different possible presentations of incarceration history in an unstructured setting, it is possible that this model would not attend well and misrepresent incarceration history in other unstructured data settings such as clinician notes outside of the ED. In addition, with each creation of definitions and annotations, these iterations themselves may add to misclassifications and further decrease the external validity of this NLP model. These misclassifications contributed to a slightly lower specificity in our Clinical-Longformer model when compared to previous YTEX model (99.3%) or ICD-10 code identification. Given the complexity of disparity in healthcare, the impact of incarceration, and stigma surrounding incarceration, any marker for incarceration history should be closely scrutinized.

While the NLP cannot overcome perceived and extant biases in the healthcare system that lead to these documentation shortcomings, our hope is that improving the ease of identifying previously incarcerated individuals for health services research and connection with community programs decreases the stigma around discussions about incarceration. Regarding our NLP itself, while it performs well, it is still early in its development. The majority of phrases used to describe incarceration have likely been captured in this model; however, there are certainly other contextual words and phrases that insinuate a history of incarceration that may have been missed and would make this model even stronger.

The Clinical-Longformer NLP was limited in its ability to distinguish the temporal relationship of incarceration based on individual unstructured ED notes. Individually, current, recent, and prior history of incarceration labels were relatively poor in identification compared to identification of “any” prior history of incarceration. Temporal relationships not specific to incarceration have been shown to be difficult to extract using current NLP frameworks [25]. This framework, dependent upon using text from unstructured clinical text taken from a specific time frame, structurally limits the ability for the NLP to extract relevant information to establish temporal relationships. Regarding GPT-4, other research has shown ChatGPT also demonstrates difficulty in identifying temporal between two events. However, ChatGPT has exhibited strong performance in causal relationships and discourse connectives in other work. It is possible that these other forms of evaluation, given enough context within the clinical notes, are allowing ChatGPT to demonstrate superior identification in the temporal labels of prior, recent, and current incarceration through means not necessarily dependent on pure temporal reasoning. The Clinical-Longformer NLP model was not able to distinguish the temporal relationship of incarceration history based on each individual clinician note as well as GPT-4, but it was still superior in its identification of any history of incarceration. The identification of any history of incarceration, however, is still important in its own right regardless of recency as the very exposure to incarceration is correlated with a wide array of adverse health conditions such as greater self-reported chronic conditions, infectious disease, and mortality [31].

Our NLP model serves as a proof-of-concept for future projects aimed at using machine learning to utilize the vast amount of information present in EHR to provide targeted interventions and treatment to patients. Further, improving the ability of this NLP model to attend across multiple notes across data available longitudinally can possibly improve the usage of this model in stratifying incarceration history into distinct sub-periods. The Clinical Longformer here was measured against a dataset of 1000 manually annotated notes based on definitions developed thorough literature review and consultation with experts that was iteratively performed to ensure consistent and reliable annotation. Future application could include measuring this NLP model using linked data systems including EHR and DOC systems.

## Conclusions

Our NLP model utilizing Clinical-Longformer with a semi-supervised machine learning approach represents both a reliable and accurate method for identifying incarceration status from nonstructured free form clinician notes in an EHR. It presents several advantages over other methods of identification of incarceration history, such as ICD-10 codes, simple keyword searches, including greater sensitivity specificity [19]. Future research can continue to fine-tune this tool, potentially allowing for the differentiation of current versus previous incarceration in order to better target services and interventions offered to these individuals.

## Supporting information

Huang et al. supplementary material Huang et al. supplementary material

## Supplementary material

The supplementary material for this article can be found at https://doi.org/10.1017/cts.2024.496.
