# Supplementary material for: Identifying incarceration status in the electronic health record using large language models in emergency department settings
Source: J Clin Transl Sci. 2024 Mar 11;8(1):e53. doi: 10.1017/cts.2024.496 (PMC10966832; doi:10.1017/cts.2024.496)
Supplement: Huang et al. supplementary material [file S2059866124004965sup001.docx]

# Appendix

**Appendix A**

Frequency table of each note type within the 1,000 annotated notes taken from the ED setting.

| **Note Type** | **Count** |
| --- | --- |
| ED Provider Notes | 462 |
| ED Notes | 120 |
| Progress Notes | 94 |
| Plan of Care | 88 |
| ED Psychiatric Eval Note | 55 |
| Consult Note | 40 |
| H&P | 35 |
| Admission / Intake | 25 |
| Discharge Summary | 17 |
| Discharge Instructions | 12 |
| ED Observation Note | 11 |
| Telephone Communication | 7 |
| SPOC-Behavioral Health | 6 |
| Consults | 6 |
| Operative Note | 5 |
| Evaluation | 4 |
| Telephone Encounter | 3 |
| ED Student Provider | 2 |
| CSC Progress Note | 2 |
| Assessment & Plan Note | 1 |
| Referral | 1 |
| Office/Comment Note | 1 |
| Group Session | 1 |
| H&P (View-Only) | 1 |
| Brief Op Note | 1 |

**Appendix B**

RoBERTa performance metrics and RoBERTa multilabel performance confusion matrix

|  | Current Incarceration | Prior History Incarceration | Recent Incarceration | macro avg | micro avg | samples avg | weighted avg |
| --- | --- | --- | --- | --- | --- | --- | --- |
| precision | 0.5625 | 0.782609 | 0.724138 | 0.689749 | 0.744526 | 0.3825 | 0.744897 |
| recall | 0.5625 | 0.757895 | 0.65625 | 0.658882 | 0.713287 | 0.3825 | 0.713287 |
| f1-score | 0.5625 | 0.770053 | 0.688525 | 0.673693 | 0.728571 | 0.375667 | 0.728586 |
| support | 16 | 95 | 32 | 143 | 143 | 143 | 143 |

**Appendix C**

Longformer performance metrics and Longformer multilabel performance confusion matrix

|  | Current Incarceration | Prior History Incarceration | Recent Incarceration | macro avg | micro avg | samples avg | weighted avg |
| --- | --- | --- | --- | --- | --- | --- | --- |
| precision | 0.647059 | 0.849315 | 0.7 | 0.732125 | 0.783333 | 0.3475 | 0.793272 |
| recall | 0.6875 | 0.652632 | 0.65625 | 0.665461 | 0.657343 | 0.3525 | 0.657343 |
| f1-score | 0.666667 | 0.738095 | 0.677419 | 0.69406 | 0.714829 | 0.345 | 0.716525 |
| support | 16 | 95 | 32 | 143 | 143 | 143 | 143 |
